# Sevoflurane improves respiratory mechanics and gas exchange in a case series of infants with severe bronchiolitis‐induced acute respiratory distress syndrome

**DOI:** 10.1002/ccr3.1490

**Published:** 2018-03-25

**Authors:** Mirco Nacoti, Jacopo Colombo, Oliviero Fochi, Daniele Bonacina, Francesco Fazzi, Giacomo Bellani, Ezio Bonanomi

**Affiliations:** ^1^ Department of Anesthesia and Intensive Care Pediatric Intensive Care Unit Papa Giovanni XXIII Hospital Bergamo Italy; ^2^ Intensive Care Unit Department of Intensive Care Medicine Ente Ospedaliero Cantonale Ospedale Regionale Bellinzona e Valli Bellinzona Switzerland; ^3^ Department of Emergency and Intensive Care San Gerardo Hospital Monza Italy; ^4^ Department of Medicine School of Medicine and Surgery University of Milan‐Bicocca Monza Italy

**Keywords:** AnaConDa, halogenate, lung compliance, mechanical ventilation, pediatric ARDS

## Abstract

This report describes the successful use of a new intervention to improve respiratory mechanics and gas exchange in a relatively homogeneous group of infants with severe bronchiolitis‐induced PARDS after failure of conventional treatment. These results may open a new interesting area of research and management for PARDS patients.

## Introduction

Bronchiolitis is the leading cause of lower respiratory tract infections (LRTIs) and hospitalization in children less than one year old: one in three infants develops clinical bronchiolitis in the first year of life and 2–3% of these infants require hospital admission 1, mainly between 30 and 60 days of age 1, 2. Children are usually admitted to the hospital for supportive care (oxygen and hydration) until clinical recovery has taken place. However, 5–30% of hospitalized children require noninvasive or invasive ventilation and may develop pediatric acute respiratory distress syndrome (PARDS) 3 characterized by intraluminal obstruction and air trapping leading to localized atelectasis that makes conventional ventilation difficult 4. In these situations, high frequency oscillatory ventilation (HFOV) and/or extracorporeal life support (ECLS) may be necessary 5. Unfortunately, these rescue therapies have major side effects 6, 7.

Halogenated agents, thanks to their powerful bronchodilatory and sedative effects, have been used as a last resort for the treatment of life‐threatening asthma in the adult and pediatric population 8, 9, 10, 11, 12, 13. Among halogenates, sevoflurane provides faster induction and recovery, lower accumulation and causes minimal cardiac depression 14.

We hypothesized that sevoflurane may have a role in children affected by severe bronchiolitis‐induced PARDS where conventional mechanical ventilation fails and escalation in respiratory support is needed.

## Materials and Methods

We reviewed the PROSAFE (promoting patient safety and quality improvement in critical care) electronic case report form (an evolution of the Margherita electronic form 15) of children affected by severe bronchiolitis‐induced PARDS, who were refractory to conventional mechanical ventilation and required were treated with sevoflurane, before an escalation respiratory support with HFO or ECLS, from November 2015 to April 2016 in the PICU of a tertiary hospital in Bergamo, Italy.

All children were ventilated in pressure‐regulated volume control (PRVC) mode with Servo‐I ventilators (Maquet Critical Care AB, Solna, Sweden) with low‐volume ventilation (tidal volume 6–8 mL/kg) before sevoflurane therapy.

Deep sedation (modified comfort scale 6–10 point) 16, evaluated before paralysis, was guaranteed initially by continuous intravenous infusion of midazolam, ketamine and fentanyl (intravenous phase) and subsequently by sevoflurane inhalation (sevoflurane phase) (Table 1).

**Table 1:** Patients’ characteristics

Enrolled patients	Child 1	Child 2	Child 3	Child 4	Child 5
Age (days‐old)	62	39	40	84	44
Weight (kg)	4.7	4.4	4.8	5.6	3.9
Sex (M/F)	M	M	F	M	F
Infection (Isolated‐Virus)	Severe bronchiolitis (RSV)	Severe bronchiolitis (RSV)	Severe bronchiolitis (RSV + Bordetella pertussis)	Severe bronchiolitis (RSV+Haemophilus influenzae)	Severe bronchiolitis (RSV + adenovirus)
PIM 2	−3.55	−3.8	−5.93	−4.38	−3.4
OSI at sevoflurane initiation	9.78	9.57	9.38	8.6	16
Chest imaging at sevoflurane initiation	Bilateral new infiltrates	Bilateral new infiltrates	Bilateral new infiltrates	Monolateral new infiltrate	Bilateral new infiltrates
Hours from intubation to sevoflurane initiation	72	24	48	38	30
Sevoflurane MAC‐hours	40	12	24	80	50
Intubation days	11	9	14	8	12
PICU days	19	20	20	12	14

PIM2, pediatric index of mortality 2; OSI, oxygen saturation index; PICU, pediatric intensive care unit.

Continuous infusion of neuromuscular‐blocking agent was performed in both phases to reduce patient‐ventilator asynchrony, allowing also reliable measurements of respiratory mechanics.

During the intravenous phase, all patients received inhalational therapy with *β*‐agonists, adrenaline, steroids, and hypertonic solution.

During the sevoflurane phase the anesthetic was vaporized through anesthetic‐conserving device “AnaConDa” (Sedana Medical AB, Sundbyberg, Sweden) placed along the inspiratory limb of the breathing circuit and not at the Y‐piece to avoid an increase in dead space 12. An anesthesia gas analyzer monitor (IntelliVue Anesthetic Gas Module, Philips, Eindhoven, The Netherlands) was used for continuous measurement of end‐tidal sevoflurane concentration (et%) during administration. The goal was to maintain an et% corresponding to the minimum alveolar concentration (MAC)‐awake of sevoflurane for children younger than 1 year.

All patients had continuous electrocardiographic, noninvasive arterial pressure, and SpO2 monitoring. Renal and liver function were measured daily during gas administration. Data regarding age, weight, diagnosis, dosing and duration of sevoflurane administration, and side effects attributable to sevoflurane were extracted from the PROSAFE electronic database.

Hemodynamic parameters (systolic blood pressure, diastolic blood pressure, mean blood pressure, heart rate, central venous pressure), venous blood gas analysis, and respiratory measurements were obtained for each child both during intravenous sedation phase (30 min before sevoflurane initiation) and during sevoflurane sedation (30 min after sevoflurane initiation and once daily until sevoflurane discontinuation).

Pressure and flow curves were recorded using a dedicated software, the Servo Tracker v 3.0 (Maquet Critical Care AB, Solna, Sweden) for a better monitoring of the clinical response to the treatment.

Airway pressure values were obtained during tidal ventilation (peak inspiratory pressure (PIP)) and during inspiratory pause (pressure 1 (P1) and plateau pressure (Pplat)) and expiratory pause (total positive end‐expiratory pressure (PEEPt), intrinsic PEEP (PEEP i)).

Tidal volume was derived from flow curve.

We measured airway resistance (AW R), tissue resistance (T R) and respiratory system compliance (RS Cpl) as follows:

$$\text{AW R}=\frac{\text{PIP}-P1}{\text{Flow}}$$

$$\text{T R}=\frac{P1-\text{Pplat}}{\text{Flow}}$$

PeePi=PeeP t−External PEEP applied

$$\text{RS Cpl}=\frac{\text{Tidal Volume}}{\text{Pplat}-\text{PEEPt}}$$

Switching to volume controlled ventilation mode was needed to allow measurement of AWR and TR 17.

As a parameter of oxygenation, we used the oxygen saturation index (OSI), calculated as follows: mean airway pressure (MAP)xFiO_2_×100/SpO_2_ 3.

## Results

All five infants had a normal gestational age and did not present any comorbidity. Age of the five patients was, respectively, 62, 39, 40, 84, 44 days. Respiratory syncytial virus (RSV) infection was diagnosed by positive antigen scrub in all five children; children three, four, and five were also infected, respectively, by Bordetella pertussis, Haemophilus influenza, Adenovirus. All children developed respiratory failure within 7 days of known clinical insult and fulfilled the PALICC (Pediatric Acute Lung Injury Consensus Conference) criteria for moderate/severe PARDS 3. Patient characteristics are described in Table 1.

Sevoflurane was started after 48 h (mean) of intubation and intravenous sedation (Table 1) as a rescue therapy in children developing an elevated airway pressure with hypercapnia and acidemia in spite of curarization.

Thirty minutes before sevoflurane initiation, resistances of the respiratory system (airway and tissue), OSI, and indexed RS Cpl were measured, as reported in Table 2.

**Table 2:** Values of P plat, PEEP, ΔP, RS R, AW R, T R, RS Cpl‐i, OSI, pCO2 v, and pH v 30 min before and 30 min after sevoflurane initiation

Parameters	Child 1		Child 2		Child 3		Child 4		Child 5	
	Intraven	Sevo	Intraven	Sevo	Intraven	Sevo	Intraven	Sevo	Intraven	Sevo
P Plat (cmH_2_O)	23	17	29	22	27	26	32	25	29	26
PEEP tot (cmH_2_O)	8	7	12	11	14	14	8	7	8	8
Peep i	2	1	4	3	2	2	4	3	2	2
ΔP (cmH_2_O)	15	10	17	11	13	12	24	18	21	18
RS R (cmH_2_O/L/sec)	75	25	52	39	105	90	73	60	84	57
AW R (cmH_2_O/L/sec)	37.5	12.5	33	13	38	30	32	27	61	30
T R (cmH_2_O/L/sec)	37.5	12.5	20	26	68	60	41	33	23	23
RS Cpl‐i (mL/cmH_2_O/kg)	0.76	0.71	0.55	0.82	0.65	0.69	0.45	0.54	0.46	0.5
OSI	9.78	7.98	9.57	7.37	9.38	9.18	6.5	5.4	16	14.7
pCO_2_ v (torr)	100	75	78	64	94	83	62.7	58.7	67.8	66.5
pH v	7.2	7.3	7.3	7.38	7.25	7.3	7.29	7.34	7.11	7.26

P plat, plateau pressure; PEEP tot, total PEEP; PEEP i, intrinsic PEEP; ΔP, driving pressure. calculated as tidal volume/RS Cpl; RS R, respiratory system resistance; AW R, airway resistance; T R, tissue resistance; RS Cpl‐i, indexed respiratory system compliance; OSI, oxygen saturation index; pCO_2_ v, venous carbon dioxide partial pressure; pH v, venous pH.

In all the patients, respiratory mechanics and gas exchange improved 30 min after sevoflurane administration (Table 2). These improvements were maintained during the entire period of sevoflurane administration. Every day sevoflurane discontinuation and switching to intravenous sedation were tested. Sevoflurane was stopped when protective ventilation was possible under midazolam, ketamine, and fentanyl sedation. Sevoflurane was administered up to 80 MAC‐hours (Table 1). There were no cases of pneumothorax, hypotension or arrhythmias. Child four and five received dopamine to correct mild hypotension during sevoflurane administration. No neurological adverse effects were observed during sevoflurane administration. Child three developed an episode of self‐limiting tonic‐clonic seizures immediately after sevoflurane suspension. All the babies were successfully extubated and discharged home uneventfully with a maximum intubation duration and PICU length of stay of 14 and 20 days, respectively (Table 1). Eighteen months after hospital discharge, a phone interview was performed with the family of the five children: All of them presented a good recovery of the catch up grow and no major neurological impairment.

## Discussion

A small fraction of pediatric patients affected by bronchiolitis are refractory to conventional medical treatments (oxygen and hydration) and respiratory support (noninvasive or invasive mechanical ventilation) 18.

Rescue therapies are mandatory when mechanical ventilation is not safe because of high airway pressures, dynamic hyperinflation, pulmonary air leaks, and hemodynamic compromise 19.

From retrospective studies, it emerges that half of the children affected by lower respiratory tract infections and treated with rescue therapies experience a complication during the course of treatment, mainly bleeding, thrombosis, and hemodynamic failure 7, 18, 20. Therefore, treatment of these patients is associated with significant morbidity and mortality 1, 18, 20.

Based on promising data regarding status asthmaticus, we speculated that halogenated agents can be used as a rescue therapy in these children.

For the first time in the literature, we describe successful treatment of bronchiolitis‐induced PARDS with sevoflurane. We included five infants who had PARDS associated to elevated driving pressure and therefore were difficult to ventilate with conventional mechanical ventilation. All infants were on maximal medical therapy (B agonists, adrenaline, steroids and hypertonic solution). We observed that, immediately after sevoflurane initiation, respiratory mechanics dramatically improved allowing return to protective ventilation settings and correction of acidosis, hypercapnia, and hypoxemia. Moreover, these beneficial effects persisted throughout the continuation of sevoflurane inhalation.

To the best of our knowledge, the only previous report of sevoflurane use in bronchiolitis is by Papoff et al. 21. While in their study a nonapproved delivery‐device was used, sevoflurane concentration was not monitored and its physiological effects were not measured, in our case series, we administered sevoflurane using AnaConDa devices, measuring sevoflurane alveolar concentration and its impact on respiratory mechanics and gas exchange. Furthermore, Papoff included three patients affected by severe bronchiolitis (two of them with spontaneous breathing), while we analyzed children affected by bronchiolitis‐induced PARDS.

Despite the long duration of sevoflurane therapy, there were no major side effects. Child three presented seizures after sevoflurane discontinuation and was treated with midazolam. He did not require any further anticonvulsive treatment. Sevoflurane‐related seizures remain to be fully explored 22. Moreover, this child was coinfected by Bordetella pertussis, a recognized seizure‐inducing condition 23.

Preclinical data suggest that anesthetic and hypnotic drugs such as halogenated agents and midazolam may affect brain development 24. The GAS study 25 found no increase in adverse neurodevelopmental outcome in infants exposed to a short period of sevoflurane anesthesia. Long‐term use of sevoflurane in children and its effect on neurological development remains unclear (24).

This study was not designed to explore the mechanisms underlying the beneficial effects that sevoflurane had on these children. Nonetheless, they can be postulated analyzing its pharmacologic proprieties: sevoflurane is an inhalational anesthetic agent with powerful bronchodilation properties, and as demonstrated in status asthmaticus, it reduces/resolves severe bronchoconstriction refractory to conventional treatment 9, 10, 11. Furthermore, it provides an excellent sedation and therefore improves patient‐ventilator adaptation 14.

We postulated that the reduction of the resistances by sevoflurane may reduce the differences between time constants of the various functional units within the lung infected by RSV. This, in turn, could explain the consequent improvement in RS Cpl and gas exchange 26. Moreover, the lower airway resistances could have lessened the pathogenic impact of RSV infection 4 (characterized by intraluminal obstruction and air trapping leading to localized atelectasis) by decreasing gas trapping, as shown by the decrement in intrinsic PEEP. This, in turn, could have led to less hyperinflation and overdistension, resulting in surprisingly improved compliance and decreased driving pressure 27.

## Conclusions

We suggest to consider a “sevoflurane test” as a rescue therapeutic option in infants with severe life‐threatening bronchiolitis‐induced PARDS when conventional mechanical ventilation has failed. It yields a reduction of both components of the R_RS_ and a gas exchange improvement. Inhaled sevorane administration in the PICU with conventional ventilators through the AnaConDa system require an involvement of trained anesthetist and may be challenging and costly where PICU are not run by anesthetist. Nevertheless, a “sevorane test” before ECLS initiation seems a reasonable option.

We think these results may stimulate a new approach to research and clinical management in PARDS area.

Further studies are needed to explore safety of long‐term sevoflurane use and its effect on important clinical outcome such us PICU length of stay and long‐term respiratory status.

## Authorship

MN, DB: were responsible for the concept, design, acquisition, analysis, and interpretation of data and drafting. JC: analyzed and interpreted the data, drafted the manuscript, and critically revised the article. FF, OF, GB: were involved in interpretation of the data, and critical revision of the article. EB: was responsible for concept, design, and critical revision of the article.

## Conflict of Interest

None declared.
